# Causal relationship between telomere length and sepsis: a bidirectional Mendelian randomization study

**DOI:** 10.1038/s41598-024-56205-z

**Published:** 2024-03-05

**Authors:** Jiamin Xu, Gehua Zhu, Hongyan Zhang

**Affiliations:** Medical Center of Burn Plastic and Wound Repair, The First Affiliated Hospital, Jiangxi Medical College, Nanchang University, Nanchang, China

**Keywords:** Risk factors, Clinical genetics, Infection, Genetics research

## Abstract

Numerous observational studies have elucidated a connection between leukocyte telomere length (LTL) and sepsis, yet its fundamental cause remains enigmatic. Thus, the current study’s objective is to employ a bidirectional Mendelian randomization (MR) approach to scrutinize the causality between LTL and sepsis. We selected single nucleotide polymorphisms (SNPs) associated with LTL (n = 472,174) and sepsis from a genome-wide association study (GWAS), including Sepsis (n = 486,484, ncase = 11,643), Sepsis (28 day death in critical care) (n = 431,365, ncase = 347), Sepsis (under 75) (n = 462,869, ncase = 11,568), Sepsis (28 day death) (n = 486,484, ncase = 1896), and Sepsis (critical care) (n = 431,365, ncase = 1380), as instrumental variables (IVs). The inverse variance weighted (IVW) MR method was employed as the primary approach, and various sensitivity analyses were conducted to assess the validity of this instrument and potential pleiotropy. Using the IVW method, we uncovered a potential causal relationship between genetically predicted LTL reduction and increased susceptibility to sepsis, with an odds ratio (OR) of 1.161 [95% confidence interval (CI) 1.039–1.297, p = 0.008]. However, reverse MR analysis did not indicate any impact of sepsis on LTL. Our forward MR study highlights a potential causal relationship between LTL as an exposure and increased susceptibility to sepsis. Specifically, our findings suggest that individuals with genetically determined shorter LTL may be at an increased risk of developing sepsis. This may contribute to the development of novel diagnostic and therapeutic strategies for the prevention, diagnosis, and treatment of sepsis.

## Introduction

Sepsis is a potentially fatal illness that develops when the body’s reaction to infection is mismanaged and causes organ malfunction^1^. Around 90,000 fatalities attributed to sepsis resulted from an estimated 480,000 sepsis cases worldwide in 2017, according to the Institute for Health Metrics and Evaluation (IHME), a research center focusing on health metrics and evaluation^2^. Sepsis often results in the impairment of multiple organ functions throughout the body, leading to various complications. The location and kind of infection, a person’s inflammatory response, treatment efficacy, and other factors affect the severity and prognosis of sepsis. Given the high incidence and mortality rates of sepsis, enhancing its prevention, recognition, and treatment is of paramount importance^3^. Early identification of risk factors is widely acknowledged as crucial for sepsis intervention^4^.

The protective structures known as telomeres are found at the brink of linear chromosomes, including DNA repeat sequences and specific DNA-binding proteins^5^. A hallmark of biological aging is the length of the telomere^6,7^. Because the cell replication machinery cannot completely replicate the chromosome ends, each cell division results in the loss of 50–100 base pairs. Therefore, as cells age, Telomere length (TL) continuously decreases. TL has become medicine’s most widely accepted individual biological age measurement standard^8^.

Telomere shortening is associated with immune dysfunction and is, therefore, considered a marker of immunological aging^9^. Existing data suggest a close relationship between TL and inflammation and the immune system, indicating a potential connection between TL and sepsis. An animal experiment’s results have shown that after lipopolysaccharide (LPS)-induced acute lung injury (ALI), telomeres in rat heart tissue exhibited shortening, suggesting that sepsis may affect TL in the chromosomes of the animal’s heart^10^. Recent research assessed variations in leukocyte telomere length (LTL) during 7 days in 40 critically sick patients, finding that LTL shortened in 21 patients, lengthened in 11, and remained unchanged in the rest of the patients. However, this study did not establish a clear association between these changes and clinical outcomes^11^. Additionally, an observational study reported a correlation between shorter LTL and higher mortality rates, particularly in sepsis patients, among critically ill patients. This study conducted a prospective observation of 937 critically ill patients and validated it in a squad of 394 sepsis critically ill patients^12^.

**Figure 1:**
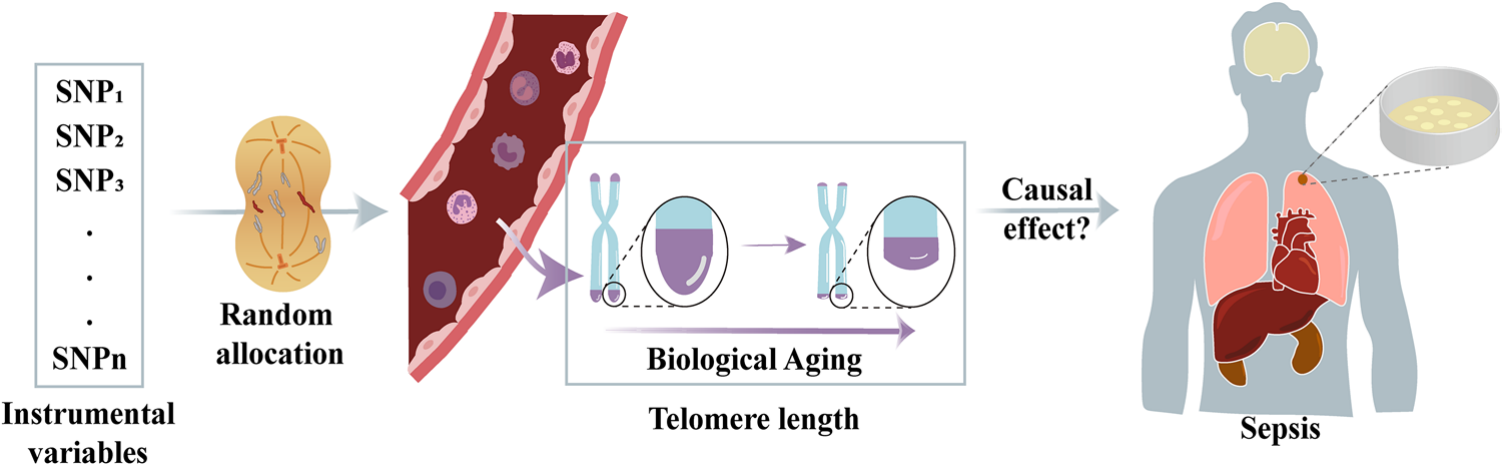
Core diagram for exploring the association between LTL and sepsis.

Increasing evidence suggests an association between LTL and sepsis. However, due to potential confounders or reverse causation, biases in the observed relationship between sepsis and LTL may exist in observational studies^13^. Therefore, whether sepsis is a cause or a consequence of telomere shortening remains unclear. Mendelian randomization (MR) can overcome these limitations; it is a robust instrumental variable (IV) method that examines causal relationships between exposure and outcome phenotypes using genetic diversity obtained from population genetics as an IV^14–16^. Single nucleotide polymorphisms (SNPs) are randomly assigned at conception, independent of confounding factors, making MR comparable to randomized controlled trials (RCTs)^17^. Additionally, individuals are largely unaware of their genotypes at the detected loci, helping to mitigate biases related to individual functional differences. Moreover, it is well-established that the pathway from genomic sequence to transcription translation into complex phenotypes is unidirectional, thus MR addresses the potential for reverse causation^18^. Our study aims to assess the bidirectional relationship between LTL and sepsis within the framework of a two-sample MR study (Fig. 1). To ensure that MR assumptions are not violated, several steps are taken. Firstly, horizontal pleiotropy analysis is conducted to account for any direct impacts of IVs on the outcome, even in the absence of the exposure of interest^19^. Secondly, SNPs in linkage disequilibrium (LD) are removed to minimize confounding by IVs associated with causal variants^20,21^. Thirdly, restricting the study population to the same racial background helps mitigate inherent differences between different populations (confounders).

## Materials and methods

### Study design

**Figure 2:**
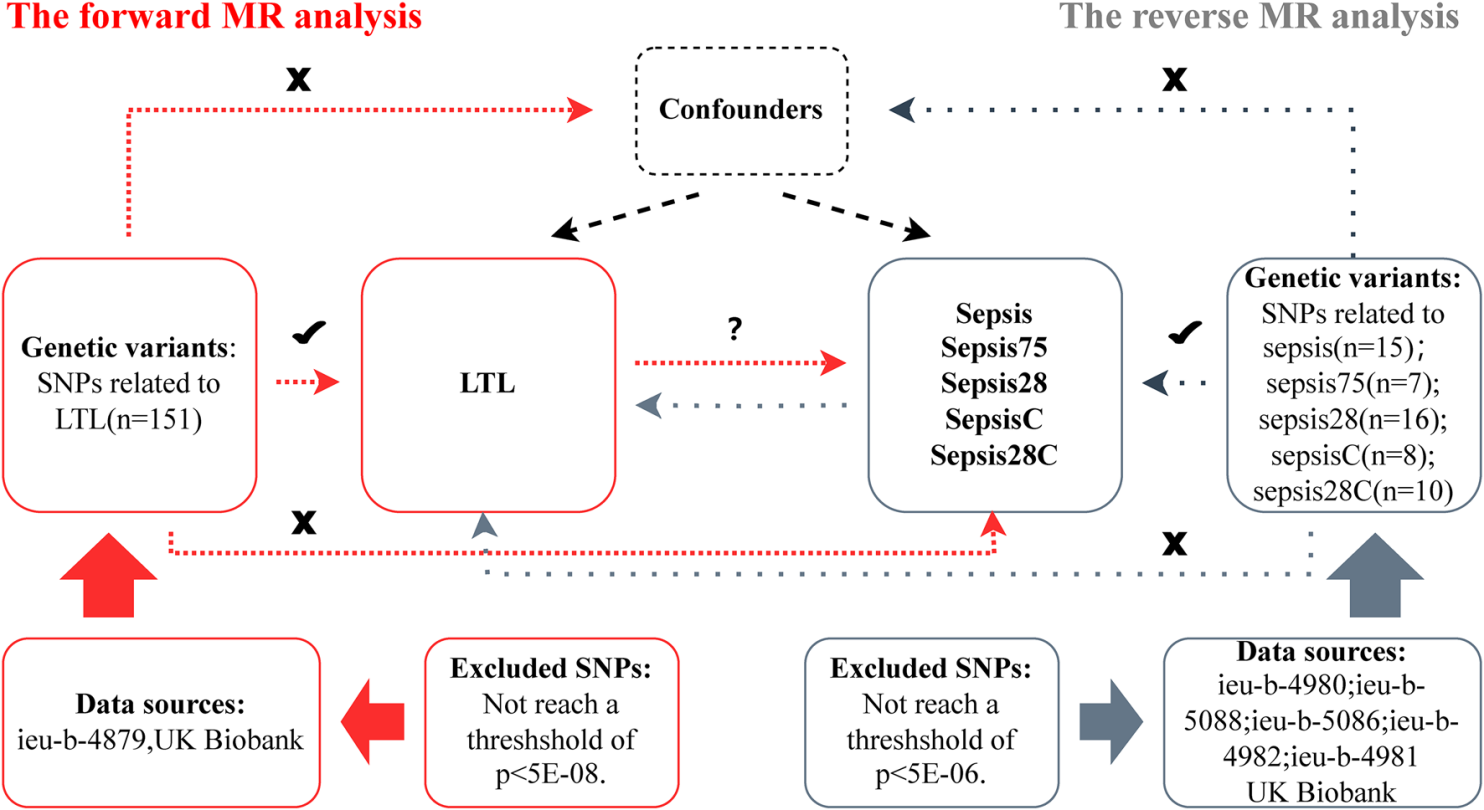
An explanation of the bidirectional MR research’s study design.

The study utilized a bidirectional MR design to investigate the correlation between LTL and sepsis, as illustrated in Fig. 2. Multiple MR analyses are conducted using summary statistics from genome-wide association studies (GWAS) to investigate the bidirectional relationship between LTL and sepsis. The forward MR analysis entailed the consideration of LTL as the exposure and sepsis as the outcome. Conversely, in the reverse MR analysis, the roles of the exposure and outcome variables were inverted. The soundness of MR analysis is predicated upon three essential suppositions: (1) the genetic variants used in the analysis should be significantly associated with the exposure; (2) the genetic variants extracted as exposure IVs should be unrelated to confounders associated with the chosen exposure and outcome; (3) genetic variants should affect the outcome only through the exposure and not through other biological pathways (i.e., no horizontal pleiotropic effects)^16,22^. The data utilized in this study originate exclusively from GWAS and publicly available data from existing literature. No novel investigations involving human or animal subjects were conducted by any authors of this study. Specifically, the UK Biobank research involving biological samples has obtained approval from the Northwest Multicenter Research Ethics Committee (Application 11/NW/0382). Participants have provided written informed consent, granting permission for the utilization of their medical records and samples for research purposes related to health. The consent statement can be found at https://www.ukbiobank.ac.uk/media/05ldg1ez/consent-form-uk-biobank.pdf^23^. Additionally, this study strictly adheres to the fundamental principles outlined in the STROBE-MR (Strengthening the Reporting of Observational Studies in Epidemiology—Mendelian Randomization), aimed at gaining a more accurate and in-depth understanding of this specific field^24^ (Supplementary Materials).

### Instrumental variable selection for MR analysis

The appropriate IVs for the MR analysis were selected from 6 different GWAS summary-level databases (Supplementary Table S1). First, SNPs were chosen based on a genome-wide significance threshold (p < 5 × 10^–8^). Next, SNPs with an LD of R^2^ < 0.001 were retained^25^. Palindromic SNPs with intermediate allele frequencies were deleted during data harmonization, while SNPs associated with the outcome with p < 5 × 10^−8^ were omitted. When harmonizing exposure and outcome data, SNPs with allele frequencies in the middle were removed. With the aid of F-statistics, we calculated the instruments’ strength. An F-value less than 10 indicates low instrument strength^26^.

### Data source and instrumental variable selection for LTL

The IVs related to LTL were extracted from the largest GWAS conducted to date, encompassing 472,174 individuals aged between 40 and 69. The gender distribution in the study was approximately equal, with 45.8% being male and 54.2% being female. Self-reported ethnicity of the participants predominantly indicated European descent (94.3%), with a minority representation of Asians (1.9%), Black individuals (1.5%), Chinese (0.3%), mixed race (0.6%), and other ethnic backgrounds (0.9%). Additionally, the study incorporated adjustments for age, gender, and ethnicity^27^. We specifically selected independent SNPs (R^2^ < 0.001) that achieved genome-wide significance (p < 5 × 10^–8^) in this GWAS as IVs for LTL and assessed the strength of each IV using F-statistics.

### Data source and instrumental variable selection for sepsis

We selected 5 GWAS datasets from the IEU OpenGWAS database, representing research on sepsis. These datasets are named sepsis, sepsis (28 day death in critical care) (sepsis28C), sepsis (critical care) (sepsisC), sepsis (28 day death) (sepsis28), and sepsis (under 75) (sepsis75). The participants in these studies are primarily of European descent, encompassing both male and female individuals. Researchers employed Regenie v2.2.4 for GWAS data analysis, adjusting for age, gender, chip type, and the first 10 principal components of the analysis (further details can be found in Supplementary Table S1)^28,29^. For the reverse MR analysis, to enhance statistical power, we chose a significance threshold of p < 5 × 10^–6^ for SNP selection, a commonly used threshold in many MR studies^30^. Furthermore, we conducted an assessment of LD (R^2^ < 0.001) and evaluated each genetic instrument’s power using the F-statistic.

### Statistical analysis

The major statistical method employed in our study was the random-effects inverse variance weighted (IVW) approach^31^, which was utilized to investigate possible bidirectional causal relationships between LTL and sepsis. The meta-analysis technique of IVW transforms the impact of the IV on exposure effects into a weighted regression, with the intercept set to zero. In the absence of horizontal pleiotropy, IVW can provide unbiased estimates by mitigating the influence of confounders^31,32^. It must be acknowledged that the effectiveness of the IVW method relies on the validity of all core assumptions of MR. If these fundamental assumptions are not met, the IVW method may introduce bias. For instance, the presence of invalid SNPs or horizontal pleiotropy can lead to bias. Therefore, we also conducted other methods such as MR-Egger, Weighted median, Weighted mode, and Simple mode to ensure precise causal estimations despite the existence of these concerns^33,34^. The MR-Egger is capable of identifying and correcting for multicollinearity, assuming that the IVs included satisfying the instrument strength independent of the direct effect (INSIDE) assumption, which posits independence between the exposure to the instrument and the association of the instrument with the outcome^35^. The weighted median method can provide precise and reliable effect estimates if at least 50% of the data from effective instruments are available^33^. For genetic instruments violating the assumption of pleiotropy, the weighted mode method proves to be adaptive. The simple mode offers robustness against pleiotropy, although its performance may not match that of the IVW method^17^.

The R^2^ value was computed to indicate the proportion of genetic variation that accounts for the exposure variable under the correlation assumption^21^. In this study, a series of sensitivity analyses were conducted to ascertain whether the heterogeneity and pleiotropy of the included IVs would introduce bias in the MR results, ensuring the robustness of our statistical findings. Cochran’s Q statistic was employed to assess the heterogeneity of the IVW and MR-Egger methods, with a p < 0.05 indicating potential heterogeneity^36^. We utilized the MR-Egger regression intercept along with its corresponding 95% confidence interval (CI) to assess and correct for potential bias arising from chance estimation errors due to horizontal pleiotropy. When horizontal pleiotropy is absent, the intercept will tend to approach zero^37,38^. Additionally, we employed the Mendelian Randomization Pleiotropy Residual Sum and Outlier (MR-PRESSO) global test and outlier test to assess horizontal pleiotropy and identify potential outliers^39^. Causal relationships between LTL and sepsis were ascertained through scatter plots, and the possibility of horizontal pleiotropy was assessed by implementing funnel plots. Finally, sensitivity analyses, including leave-one-out analyses, were conducted to determine whether individual SNPs had an impact on the main causal associations^40^.

The establishment of statistical significance was based on the criterion that the two-sided P-value was below 0.05. Statistical analyses, data output, and visualization were all performed using the “MendelianRandomization” package^41^, the “TwoSampleMR” package^42^, and the “MR-PRESSO” package^39^ in R version 4.3.0.

## Results

### Instrumental variables

Figure 2 presents the IVs used in this study. Ultimately, we identified 151 SNPs linked to LTL, 15 SNPs correlated with sepsis, 7 SNPs linked to sepsis 75, 16 SNPs associated with sepsis28, 8 SNPs connected to sepsisC, and 10 SNPs associated with sepsis28C. Additionally, we conducted a strength analysis on the IVs above, and the results indicated that the F-statistics for all SNPs exceeded 20, suggesting the robustness of this study against weak IV bias. For further details, please refer to Supplementary Table S2.

### The causal effect of LTL on sepsis

**Figure 3:**
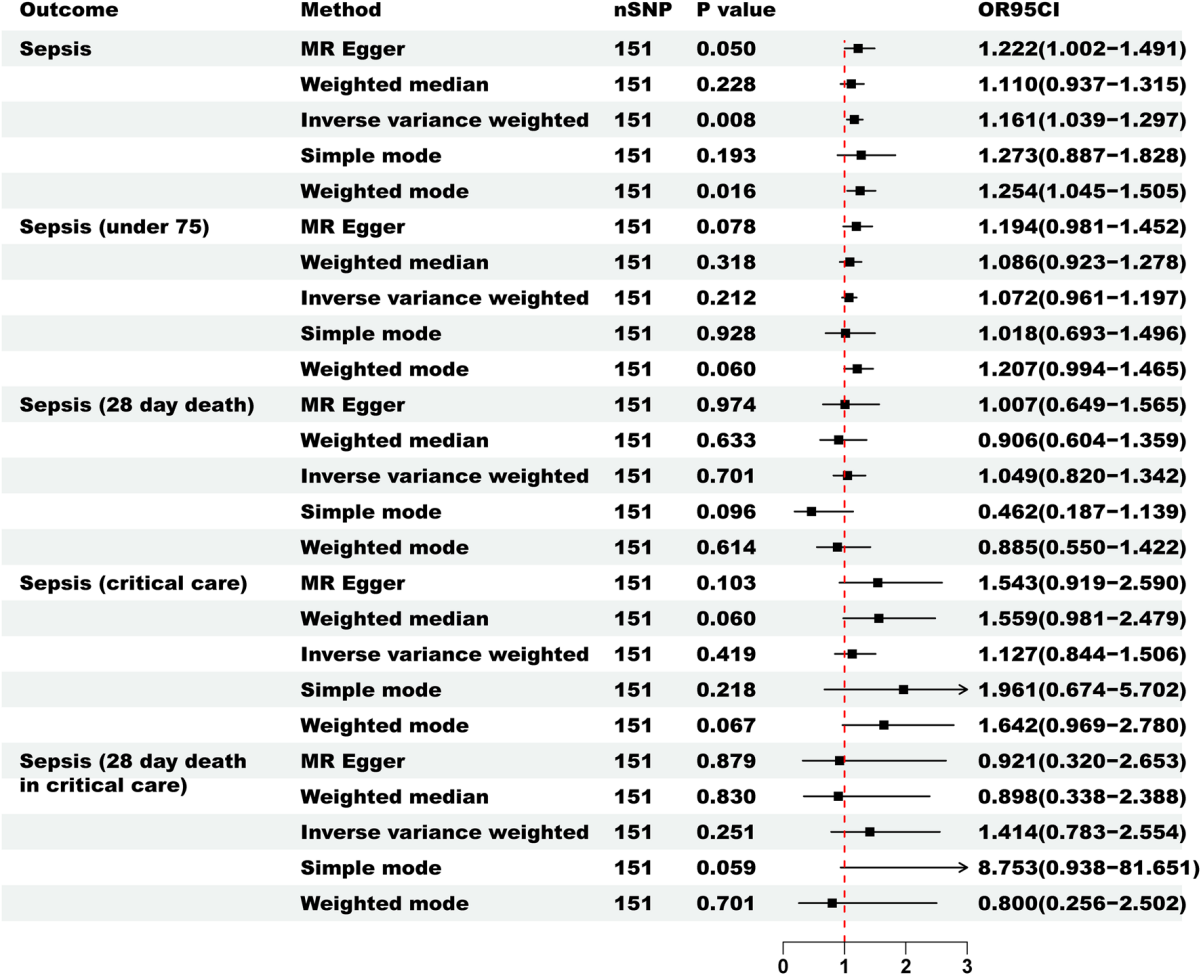
The forward MR was used to investigate the causal relationship between LTL and sepsis.

We employed the IVW causal analysis method to establish the association between LTL as an exposure and sepsis. The study findings (Fig. 3) reveal a significant correlation between LTL and susceptibility to sepsis (p = 0.008, Supplementary Table S3). Specifically, a one standard deviation decrease in genetically predicted LTL is associated with a 1.161-fold increase in the risk of sepsis [Odds Ratio (OR) 1.161 (95% CI 1.039–1.297)]. Additionally, the Weighted mode method yielded a similar estimate of the causal effect [OR 1.254 (95% CI 1.045–1.505), p = 0.016]. Although MR-Egger method [OR 1.222 (95% CI 1.002–1.491), p = 0.050], Weighted median method [OR 1.110 (95% CI 0.937–1.315), p = 0.228], and Simple mode method [OR 1.273 (95% CI 0.887–1.828), p = 0.193] all exhibited similar patterns in estimating causal effects, they lacked statistical significance in elucidating the relationship between LTL and the incidence of sepsis. In conclusion, the forward MR analysis indicates an association between genetically predicted shorter LTL and an increased incidence of sepsis.

It is worth noting that we found no significant associations between LTL and sepsis75 [OR 1.072 (95% CI 0.961–1.197), p = 0.212], sepsis28 [OR 1.049 (95% CI 0.820–1.342), p = 0.701], sepsisC [OR 1.127 (95% CI 0.844–1.506), p = 0.419], and sepsis28C [OR 1.414 (95% CI 0.783–2.554), p = 0.251]. The outcomes derived from the simple mode, weighted mode, weighted median, and MR-Egger methods demonstrate concordance with the aforementioned observations (Supplementary Table S3).

When analyzing the causal relationship between LTL and sepsis, we observed heterogeneity among the instruments for sepsis (IVW: Q = 181.215, p = 0.042; MR-Egger: Q = 180.768, p = 0.039) and sepsis75 (IVW: Q = 185.287, p = 0.027; MR-Egger: Q = 183.227, p = 0.030) as indicated by Cochran’s Q test. However, the p-values for Cochran’s Q test for sepsis28, sepsisC, and sepsis28C were all greater than 0.05, suggesting no significant heterogeneity. Additionally, we performed a comprehensive MR-PRESSO examination, which did not detect any aberrant data points. Importantly, the sensitivity analysis conducted via the MR-Egger intercept test did not reveal any discernible signals of horizontal pleiotropy (Supplementary Table S4, p > 0.05). Elaborate scatter plots, funnel plots, and leave-one-out plots pertaining to the causal effect analysis of LTL on sepsis are available in Supplementary Fig. S1. The above statistical results indicate the stability of the forward MR analysis findings.

### The causal effect of sepsis on LTL

**Figure 4:**
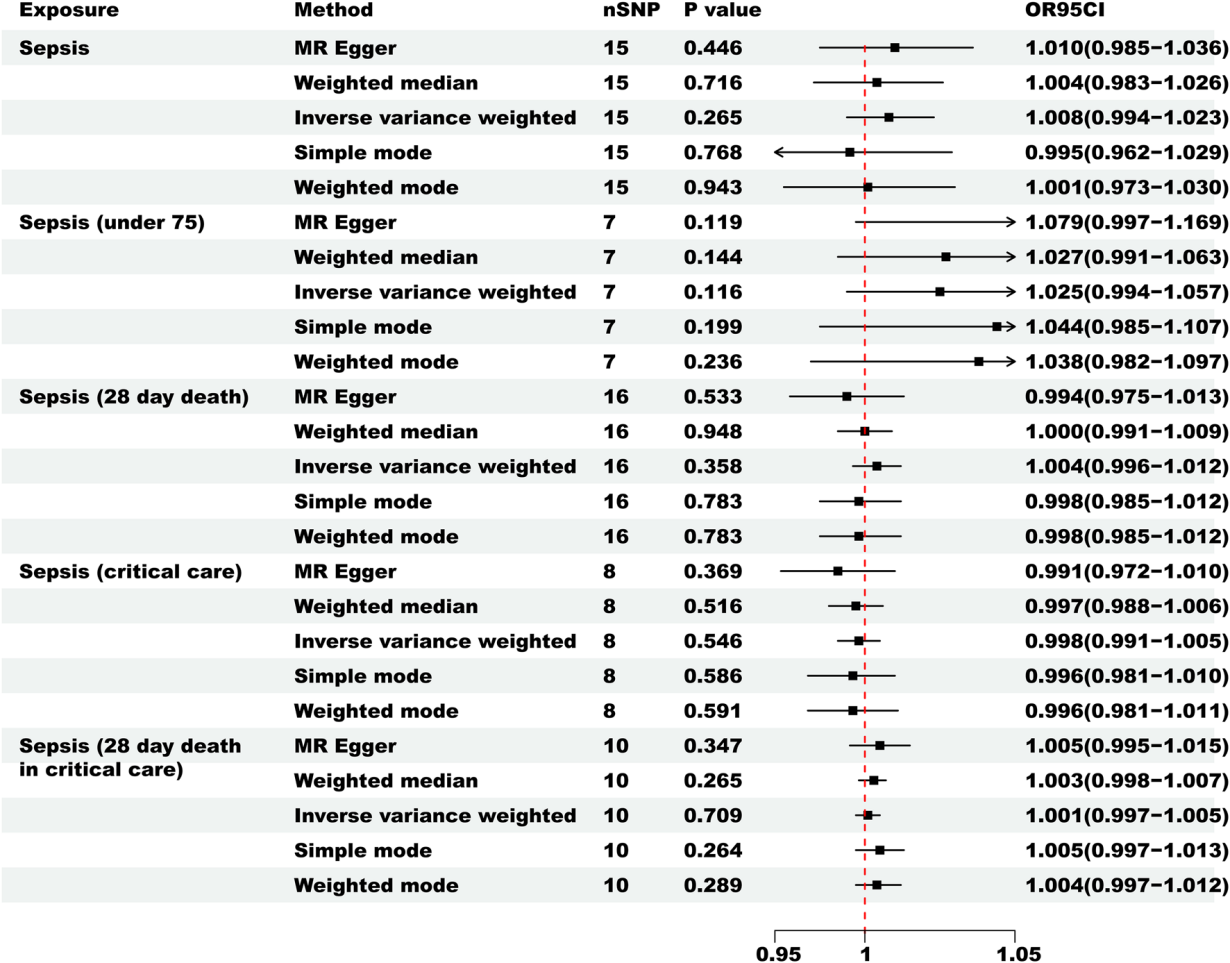
The reverse MR was used to investigate the causal relationship between sepsis and LTL.

Figure 4 depicts the results of MR analysis regarding the causal relationship between sepsis as an exposure and LTL. According to the IVW analysis results (Supplementary Table S3), there is no substantial evidence supporting a genetically predicted causal relationship between sepsis (β = 0.008, SE = 0.007, p = 0.265), sepsis75 (β = 0.025, SE = 0.157, p = 0.116), sepsis28 (β = 0.004, SE = 0.004, p = 0.358), sepsisC (β =  − 0.002, SE = 0.004, p = 0.546), and sepsis28C (β = 0.001, SE = 0.002, p = 0.709) with LTL. The outcomes obtained through the simple mode, weighted mode, weighted median, and MR-Egger methods (Supplementary Table S4, p > 0.05) all align with the IVW findings.

To further validate the robustness of our results, we conducted heterogeneity and pleiotropy analyses. Both the Cochran’s Q test and MR-Egger intercept test results showed p-values greater than 0.05, providing almost no evidence of heterogeneity and horizontal pleiotropy among different IVs. In the global MR-PRESSO test, we detected and recorded excluded outliers (Supplementary Table S4). Furthermore, even when excluding individual SNPs, the leave-one-out analysis results remained stable (Supplementary Fig. S2), indicating the robustness of the findings. Scatter plots and funnel plots are provided in the Supplementary Fig. S2.

## Discussion

An in-depth bidirectional MR analysis using the most comprehensive GWAS database was conducted to investigate the causal relation between LTL and sepsis. As per the outcomes of the forward MR analysis, a genetically forecasted reduction in LTL is linked to an elevated vulnerability to sepsis. This finding is consistent with previous observational studies. The four alternative statistical methods, apart from the IVW method, also exhibit similar patterns in estimating causal effects. Despite p-values > 0.05, we endorse the perspective proposed by Sterne JA and Davey Smith G^43^, advocating that reporting in medical research should move away from a sole focus on the significance of results and shift towards interpreting study outcomes within the context of study design and other available evidence^44^. Furthermore, it is noteworthy that no substantial correlations were detected between LTL as exposure and the subtypes sepsis75, sepsis28, sepsisC, and sepsis28C. Through sensitivity analyses, we further ensured the reliability of the study’s findings. In the reverse MR analysis, there remained a lack of definitive evidence establishing sepsis as an exposure with a causal association with LTL.

It is important to note that the Third International Consensus Definitions Workgroup defines sepsis as “life-threatening organ dysfunction caused by a dysregulated response to infection^45^”. To gain a more comprehensive understanding of the relationship between LTL and sepsis, further research is warranted. In this investigation, we utilized bidirectional MR for the inaugural occasion, with the intention of exhaustively evaluating the causal nexus between LTL and sepsis. Prior to this, several studies had explored the relationship between TL and sepsis. A prospective study has observed that most patients experiencing acute stress exhibit telomere shortening, indicating a potential association between telomere shortening and sepsis diagnosis. Furthermore, it has been noted that there is no correlation between mortality rates of intensive care unit (ICU) hospitalized patients and TL^11^. Another prospective observational study revealed that shorter TL in peripheral blood leukocytes is associated with severe acute respiratory distress syndrome (ARDS) (OR 2.5, 95% CI 1.1–6.3 per 1 kb TL decrease; p = 0.044). This study suggests that telomere dysfunction may contribute to the occurrence of critical illnesses^12^. These conclusions align with the findings of the present MR study.

In contrast, some existing traditional observational studies present conclusions inconsistent with those of this study. A prospective study conducted in 2017, involving 75,309 participants, found an association between shorter LTL and increased risk of hospitalization due to infection and pneumonia. However, the stratified analysis indicated that TL was unrelated to the risk of developing sepsis^46^. Based on the positive MR results of this study, we are inclined to believe that traditional observational studies may struggle to overcome potential confounders or reverse causation interference.

Furthermore, while investigating the relationship between LTL and sepsis, we should also focus on the role of LTL shortening in physiological and pathological processes. In fact, telomere shortening is often considered a result of oxidative damage in the context of stress response^47^. Interestingly, Yokoo et al. found that exposure to a source of vitamin C led to a reduction in telomere shortening in human skin keratinocytes^48^. Similarly, research by Tanaka and others indicated that the rate of telomere shortening in human brain cells slowed down when exposed to phosphorylated alpha-tocopherol^49^. The imbalance between oxidative stress and the antioxidant system is considered one of the contributors to the systemic inflammatory response syndrome^50^. There is an interaction between telomere shortening, oxidative stress, and inflammation, which may partially explain the pathophysiological events following sepsis.

Numerous studies have observed associations between shortened LTL and conditions such as Alzheimer’s disease^51^, atherosclerotic diseases^52^, type 2 diabetes^20^, and cancer^53^, which are also relatively common in sepsis patients. Recent research has indicated that biological age holds more therapeutic value than chronological age, as it is believed to offer a more precise prediction of health aging^6,8,54^. Given the relationship between LTL and aging, our research findings similarly substantiate an association between biological age and the risk of sepsis. Shortened LTL may potentially heighten the risk of sepsis, even when it may be unrelated to chronological age, as LTL is currently recognized as a reliable biomarker of biological age^7^. Biological age is influenced by various variables, including high-density lipoprotein (HDL) and body mass index (BMI)^9^. These factors are considered risk factors for sepsis, and a relationship between LTL and biological age has been substantiated^13,55^. Consequently, LTL may emerge as a critical component in establishing a novel sepsis risk prediction system. In other words, the link between shorter LTL and an elevated sepsis risk may be an unexpected discovery between chronic diseases, cellular aging, and immune function. While feasible strategies to directly alter LTL is currently lacking, it has been demonstrated that adopting a healthy lifestyle can improve LTL^56,57^. Causality testing provides another epidemiological piece of evidence that supports the hypothesis that shorter LTL may heighten the risk of sepsis. This discovery also suggests that integrating LTL into preventive and management strategies may contribute to a more precise prediction of an individual’s risk of sepsis. This offers a new perspective for personalized medical care and the development of public health policies, particularly in addressing health challenges associated with population aging.

It must be acknowledged that establishing a causal relationship between LTL and susceptibility to sepsis solely through observational studies is challenging. Patients with shorter telomeres may exhibit compromised infection regulation, which could increase their susceptibility to infections, offering a potential explanation for the association between the two. Shortened LTL may be accompanied by genomic instability, reduced cell proliferation, decreased production of T lymphocytes and B lymphocytes due to cellular aging, and adverse effects on immune function, all of which might contribute to an elevated risk of sepsis. Additionally, this could lead to a cytokine storm, exacerbating sepsis and further shortening telomeres. All of these factors have been widely discussed in the literature previously^46,58,59^. Another explanation lies in the existence of distinct white blood cell subpopulations within sepsis patients, which may impact the analysis of total TL due to changes in white blood cell proportions. It is important to note that TL changes depend on the rate of replication, and whether TL found in white blood cells can represent TL in other organ tissues remains unclear^46,60^. Consequently, mitigating the introduction of confounders has been a challenge in all prior observational studies. In contrast, the research design of this study is superior, as it can leverage MR methods to demonstrate certain causal relationships, thereby reducing the potential for bias.

Based on the outcomes of the reverse MR analysis, it was ascertained that there exists no significant causal link between sepsis as an exposure and LTL. A recent prospective study assessed changes in TL in critically ill patients on the first and seventh days of admission, involving 40 critically unstable patients. Nonetheless, this investigation did not succeed in illustrating a notable correlation between alterations in TL and any outcome measures^11^. This finding further reinforces the conclusions drawn from our retrospective MR analysis. While prospective studies afford observations of TL alterations in actual patients, the results fail to demonstrate a significant association between these changes and specific clinical outcomes. This may suggest that the impact of sepsis on LTL is subject to modulation by other factors or potential confounding influences.

In our research, bidirectional MR has been employed. In comparison to conventional observational studies, its principal advantage lies in the emulation of the observational environment of randomized controlled trials. Although randomized controlled trials have gained widespread acceptance, their implementation is often costly and challenging, potentially entailing ethical concerns. MR effectively circumvents confounding bias introduced by random SNP allocation during pregnancy and mitigates the influence of reverse causality. Additionally, we utilized publicly available large-scale genomic data in our study, enhancing the stability and generalizability of the research outcomes. Furthermore, employing MR methodology allows us to assess the magnitude of causal effects, potentially influencing clinical and public health decision-making significantly. However, we acknowledge certain inherent limitations in this study. Firstly, the data primarily stem from participants of European descent, which could introduce bias for other racial and ethnic populations. To enhance the generalizability of our research findings, further validation with more diverse population samples is imperative. Secondly, in forward MR analysis, the Cochran’s Q statistic reveals that heterogeneity remains significantly high, and manual checks of instrument validity using Phenoscanner have not been conducted. Although the results from all methods are reliable, potential bias may persist due to limited genetic resources or sample overlap between exposures and outcomes. Finally, this study can only partially elucidate the pathogenic role of LTL on sepsis, as LTL is influenced by a confluence of factors, including genetics, environment, lifestyle, and epigenetics, all of which need consideration in the study.

In summary, our study utilizes the MR technique to illuminate the causal connection between LTL and sepsis, showcasing its unique advantages. Despite the presence of certain limitations, such as the bias in the racial composition of the data source, our research provides a fresh perspective for understanding the biological significance of LTL and its potential clinical relevance. Future research endeavors should be dedicated to overcoming the aforementioned limitations, including but not limited to enhancing the external validity and representativeness of studies by acquiring larger sample sizes. Additionally, a more comprehensive consideration of various factors is imperative to minimize confounding variables. The objective of this research direction is to deepen our understanding of the fundamental relationship between LTL and sepsis. Through this in-depth analysis, our aim is to offer novel diagnostic and therapeutic strategies for the prevention and treatment of sepsis.

## Conclusions

This study employed bidirectional MR analysis, with the forward MR analysis establishing a potential causal relationship between LTL as exposure and susceptibility to sepsis. Specifically, it suggests that genetically predicted shorter LTL may increase susceptibility to sepsis. The reverse causality hypothesis, however, is not supported. To gain a deeper understanding of the causal interplay between LTL and sepsis, as well as the underlying mechanisms involved, further extensive research is imperative.

### Supplementary Information

Supplementary Information.

Supplementary Figures.

Supplementary Tables.

## Data Availability

The original contributions presented in the study are included in the article/Supplementary Material, further inquiries can be directed to the corresponding author.

## Abbreviations

CI: Confidence interval
GWAS: Genome-wide association studies
IV: Instrumental variable
IVW: Inverse variance weighted
LTL: Leukocyte telomere length
LD: Linkage disequilibrium
MR: Mendelian randomization
MR-PRESSO: Mendelian Randomization Pleiotropy Residual Sum and Outlier
OR: Odds Ratio
SNP: Single nucleotide polymorphisms
Sepsis75: Sepsis (under 75)
Sepsis28: Sepsis (28 day death)
SepsisC: Sepsis (critical care)
Sepsis28C: Sepsis (28 day death in critical care)
TL: Telomere length
