# Supplementary material for: Causal relationship between telomere length and sepsis: a bidirectional Mendelian randomization study
Source: Sci Rep. 2024 Mar 5;14:5397. doi: 10.1038/s41598-024-56205-z (PMC10914758; doi:10.1038/s41598-024-56205-z)
Supplement: Supplementary file 2 — Supplementary Figures. [file 41598_2024_56205_MOESM2_ESM.docx]

***Supplementary Figure***

## 1 Supplementary Figure S1. Scatter plots, funnel plots, and leave-one-out plots between LTL and sepsis.

## 2 Supplementary Figure S2. Scatter plots, funnel plots, and leave-one-out plots between sepsis and LTL.

## 1 Supplementary Figure S1. Scatter plots, funnel plots, and leave-one-out plots between LTL and sepsis.





A, B and C represent three diagrams between LTL and Sepsis.

D, E and F represent three diagrams between LTL and Sepsis (28 day death).

G, H and I represent three diagrams between LTL and Sepsis (critical care).

J, K and L represent three diagrams between LTL and Sepsis (28 day death in critical care).

M, N and O represent three diagrams between LTL and Sepsis (under 75).

## 2 Supplementary Figure S2. Scatter plots, funnel plots, and leave-one-out plots between sepsis and LTL.





A, B and C represent three diagrams between Sepsis and LTL.

D, E and F represent three diagrams between Sepsis (28 day death) and LTL.

G, H and I represent three diagrams between Sepsis (critical care) and LTL.

J, K and L represent three diagrams between Sepsis (28 day death in critical care) and LTL.

M, N and O represent three diagrams between Sepsis (under 75) and LTL.
